# Supplementary material for: Factors accelerating time to death among persons with Tuberculosis in Western India: Evidence from a community-based retrospective death audit
Source: PLoS One. 2026 Feb 27;21(2):e0343271. doi: 10.1371/journal.pone.0343271 (PMC12948073; doi:10.1371/journal.pone.0343271)
Supplement: S1 File — (DOCX) [file pone.0343271.s001.docx]

Additional file 1

Supplementary Data are included here in additional file 1 which have been used for description of results, providing granular detail beyond the manuscript's core presentation.

Table 1 Frequency Distribution Socio Demographic Profile (N=149)

| **Variable** | **Category** | **Frequency** | **Percent** |
| --- | --- | --- | --- |
| Age Distribution (yrs) | 0 -18 years | 3 | 2 |
|  | 19-25 years | 10 | 6.7 |
|  | 26-50 years | 60 | 40.3 |
|  | 51-65 years | 49 | 32.9 |
|  | More than 65 years | 27 | 18.1 |
|  | Total | 149 | 100 |
| Gender | Female | 27 | 18.1 |
|  | Male | 122 | 81.9 |
|  | Total | 149 | 100 |
| Type of Family | Joint | 68 | 45.6 |
|  | Nuclear | 81 | 54.4 |
|  | Total | 149 | 100 |
| Education | Graduate & above | 3 | 2 |
|  | Graduate and above | 1 | 0.7 |
|  | Illiterate | 62 | 41.6 |
|  | Primary | 60 | 40.3 |
|  | Secondary & Higher Secondary | 23 | 15.4 |
|  | Total | 149 | 100 |
| Occupation | Daily Laborer | 55 | 36.9 |
|  | Employed (Govt.) | 12 | 8.1 |
|  | Employed (Private) | 7 | 4.7 |
|  | Farmer/Cultivator | 31 | 20.8 |
|  | Housewife | 16 | 10.7 |
|  | Other-specify | 8 | 5.4 |
|  | Self-employed | 16 | 10.7 |
|  | Student | 4 | 2.7 |
|  | Total | 149 | 100 |

Table 2 Frequency distribution of risk profile (N = 149)

| **Variable** | **Category** | **Frequency** | **Percent** |
| --- | --- | --- | --- |
| Key Population | No | 55 | 36.9 |
|  | Yes | 94 | 63.1 |
|  | Total | 149 | 100 |
| History of Comorbidities | No | 77 | 51.7 |
|  | Yes | 72 | 48.3 |
|  | Total | 149 | 100 |
| History of Addiction | No | 51 | 34.2 |
|  | Yes | 98 | 65.8 |
|  | Total | 149 | 100 |

Table 3 Healthcare-Seeking Behaviour of deceased persons with tb (n=149)

| **Variable** | **Category** | **Frequency** | **Percent** |
| --- | --- | --- | --- |
| Type of First Contact Health facility | PUBLIC PHI | 71 | 47.8 |
|  | PVT PHI | 47 | 31.6 |
|  | AYUSH | 15 | 10 |
|  | Don’t know | 14 | 9.3 |
|  | Local Healers | 2 | 1.3 |
|  | Total | 149 | 100% |
| No. of Health Facility Visited before confirmed diagnosis of TB | Don’t Know | 19 | 12.8 |
|  | One | 60 | 40.3 |
|  | Two | 30 | 20.1 |
|  | Three | 17 | 11.4 |
|  | More than three | 23 | 15.4 |
|  | Total | 149 | 100 |

Table 4 TB case characteristics related clinical profile (n = 149)

| **Variable** | **Category** | **Frequency** | **Percent** |
| --- | --- | --- | --- |
| Site of Diseases | Extrapulmonary | 17 | 11.4 |
|  | Pulmonary | 132 | 88.6 |
|  | Total | 149 | 100 |
| Type of TB Case | New | 96 | 64.4 |
|  | PMDT | 13 | 8.7 |
|  | Retreatment | 40 | 26.8 |
|  | Total | 149 | 100 |
| Microbiologically Confirmed | No | 49 | 32.9 |
|  | Yes | 100 | 67.1 |
|  | Total | 149 | 100 |
| Regimen | FDC | 136 | 91% |
|  | Longer Oral MDR/XDR-TB Regimen | 2 | 1.3% |
|  | MDR_FQ | 1 | 0.7% |
|  | MDR_SHORT | 6 | 4.0% |
|  | Oral_Longer_MDR+/-FQ_SLI | 2 | 1.3% |
|  | Shorter Oral MDR/RR-TB Regimen | 2 | 1.3% |
|  | Total | 149 | 100 |
| Registered Adherence tool | Directly observed treatment through Treatment Supporter | 89 | 59.7 |
|  | ICT based 99DOTS | 59 | 39.6 |
|  | MERM | 1 | 0.7 |
|  | Total | 149 | 100 |
| Adherence to the treatment | Completely Adherent | 94 | 63.1 |
|  | Non-Adherent | 55 | 36.9 |
|  | Total | 149 | 100 |

List of Abbreviations

| **Abbreviation** | **Full Form** | **Source Context** |
| --- | --- | --- |
| **yrs** | years | Age Distribution |
| **JOINT** | Joint Family | Type of Family |
| **NUCLEAR** | Nuclear Family | Type of Family |
| **Govt.** | Government | Employed (Govt.) |
| **COPD** | Chronic Obstructive Pulmonary Disease | Type of Key Populations |
| **HIV** | Human Immunodeficiency Virus | Type of Key Populations |
| **TB** | Tuberculosis | Type of Key Populations |
| **PHI** | Public Health Institution | Type of First Contact Health facility (PUBLIC PHI) |
| **PVT PHI** | Private Health Institution | Type of First Contact Health facility |
| **AYUSH** | Ayurveda, Yoga and Naturopathy, Unani, Siddha, and Homeopathy (Traditional Medicine System) | Type of First Contact Health facility |
| **PMDT** | Programmatic Management of Drug-resistant Tuberculosis | Type of TB Case |
| **FDC** | Fixed Dose Combination | Regimen |
| **MDR/XDR-TB** | Multidrug-resistant Tuberculosis / Extensively drug-resistant Tuberculosis | Regimen |
| **MDR_FQ** | Multidrug-resistant, Fluoroquinolone | Regimen |
| **MDR_SHORT** | Multidrug-resistant, Short Course Regimen | Regimen |
| **MDR/RR-TB** | Multidrug-resistant Tuberculosis / Rifampicin-resistant Tuberculosis | Regimen |
| **ICT** | Information and Communications Technology | Registered Adherence tool (ICT based 99DOTS) |
| **MERM** | Medication Event Reminder Monitoring | Registered Adherence tool |
