# Supplementary material for: Factors accelerating time to death among persons with Tuberculosis in Western India: Evidence from a community-based retrospective death audit
Source: PLoS One. 2026 Feb 27;21(2):e0343271. doi: 10.1371/journal.pone.0343271 (PMC12948073; doi:10.1371/journal.pone.0343271)
Supplement: Attachment — Submitted filename: Reviewer Comments_PONE-D-25-61087.docx [file pone.0343271.s002.docx]

**Reviewer Report**

**Manuscript Title:**

A Cross-Sectional Study on Factors Accelerating Time-to-Death Among Individuals Notified with TB: Evidence from a Community-Based Death Audit in Western India

**Reviewer Comments:**

Overall, the manuscript addressed an important public health issue of early mortality among tuberculosis patients (PwTB) in a high-burden Indian state. The use of community-based verbal autopsy (CBVA) linked with timeline and Cox regression analysis offers meaningful insights for TB mortality surveillance. The study is relevant to the Indian context, aligned well with the policy and can be good evidence for Tuberculosis related literature. However, some methodological and structural issues need be addressed to strengthen the manuscript for publication.

1. The study is described as cross-sectional, but the analytical approach like time-to-event, Cox model and timelines from longitudinal data need to be aligned with traditional cross-sectional definitions. The study seems to be a "retrospective cohort using verbal autopsy data". Please justify its classification as cross-sectional study.
2. The sampling frame is unclear as it says that data was taken from "Quarterly notification registers 2023–24," but verbal autopsies include deaths from 2022–2023. Inclusion period appears inconsistent. Please specify clear timelines of notification period, death period, Interview-verbal autopsy period.
3. Based on >5% death reporting, the study districts were purposively selected. This may overestimate mortality-related determinants. Comparison is needed with the baseline population of all TB deaths or general PwTB. A brief justification is needed about why these 6 districts were selected and do they adequately represent Gujarat. Comment about potential selection bias and its effect on generalizability.
4. Please detail about the Reliability and Validity of Verbal autopsy tool as modifications were done in WHO tool. Please specify what items were adapted, whether tool was validated locally, inter-rater reliability assessments of data collectors.
5. Please discuss about potential confounders and how they were adjusted in the Cox model. acknowledge the unmeasured confounders.
6. Discuss about how the recall bias was mitigated.
7. Results: Table 3 lacks key diagnostics such as Number of events per variable, Model fit indices, Multicollinearity tests.
8. Discussion: While associations are strong, the discussion often implies causality. Modify the language suitably. Avoid implying predictive models unless validated.
9. TBDSR recommendations are relevant but require links to recent state/national policy efforts. Shorten and cite examples from West Bengal, South Africa, or WHO TB mortality audits.
10. Several sentences are long or repetitive. Please maintain consistent terms: PwTB / TB patients / deceased individuals.
11. Ethical Statements: Two contradictory ethical statements can be seen, one in manuscript body and one in declarations section with different approval numbers and dates. Please recheck and correct.
12. Reference formatting is inconsistent. Please modify as per journal guidelines.

**Recommendation:**

**Major Revision**

The manuscript presents important findings but requires substantial revisions in methodology, reporting standards, results and discussion.
