# Supplementary material for: Factors accelerating time to death among persons with Tuberculosis in Western India: Evidence from a community-based retrospective death audit
Source: PLoS One. 2026 Feb 27;21(2):e0343271. doi: 10.1371/journal.pone.0343271 (PMC12948073; doi:10.1371/journal.pone.0343271)
Supplement: Attachment — Submitted filename: Reviewer Comments_PLoS.docx [file pone.0343271.s004.docx]

**Manuscript Title**

**Factors Accelerating Time to Death Among Persons with Tuberculosis in Western India: Evidence from a Community-Based Retrospective Death Audit**

**Manuscript ID**

PONE-D-25-61087R1

**General Comments**

This study has addressed an important policy relevant issue of early mortality among persons with tuberculosis (PwTB) in India, as India bears the highest global TB burden. TB mortality remains underexplored compared to incidence and treatment outcomes; this study directly addresses a critical gap. The researchers have used community-based verbal autopsy data to examine clinical, social, and health-system determinants of accelerated mortality. This method is well-aligned with national priorities under the National Tuberculosis Elimination Programme (NTEP). The adaptation of the WHO verbal autopsy tool to capture TB care-cascade timelines is a major strength. Cross-verification with Nikshay and treatment records improves credibility and minimizes recall bias. Findings are highly relevant to India’s TB elimination goals and comparable high-burden settings. The revised version demonstrates significant improvements as per editor suggestions. Overall, the manuscript is methodologically sound, very well written, and offers actionable insights for TB mortality surveillance and response systems. The manuscript would be suitable for publication, with a few minor corrections.

**Strengths:**

1. The use of Cox proportional hazards modeling, supported by diagnostic checks (Schoenfeld test, VIF), is appropriate for evaluating factors associated with shortened survival intervals.
2. Presentation of median-based timelines is statistically justified given the skewed distribution of delay data.
3. Identification of key population status, comorbidities, and drug-resistant TB as drivers of early mortality provides clear targets for intervention.
4. The discussion around TB Death Surveillance and Response (TBDSR) is aligned with WHO and national frameworks.
5. Ethical approval, consent procedures, and data availability statements are clearly described and consistent.
6. Reporting aligns with PLOS ONE standards for observational studies and use of human participant data.

**Suggested Minor Corrections:**

1. In study design, the justification for describing the study as a “cross-sectional audit with time-to-event analysis” is well explained. But for readers from epidemiology backgrounds, this study sounds as a retrospective cohort of deceased individuals. Consider briefly acknowledging this overlap explicitly in the Methods or Limitations to avoid conceptual confusion.
2. District selection was purposive and based on higher TB mortality (>5%), which is appropriate for identifying system bottlenecks. Authors need to reinforce that findings are most applicable to high-mortality or high-risk programmatic settings.
3. Important variables such as nutritional status (BMI), severity at diagnosis, and treatment of comorbidities could not be included. Justification for these omissions is needed and briefly reiterate this point when interpreting Cox model findings to prevent overinterpretation.
4. Figure 1 is informative but dense. Please ensure that axis labels and legends are clearly readable in the final typeset version.

**Recommendation**

**Minor Revision / Accept after Minor Revisions**

The manuscript makes a meaningful contribution to the literature on TB mortality and provides actionable insights for programmatic response. Following minor clarifications as noted above, it is suitable for publication.
