# Supplementary material for: Factors accelerating time to death among persons with Tuberculosis in Western India: Evidence from a community-based retrospective death audit
Source: PLoS One. 2026 Feb 27;21(2):e0343271. doi: 10.1371/journal.pone.0343271 (PMC12948073; doi:10.1371/journal.pone.0343271)
Supplement: Attachment — Submitted filename: Rebuttal Letter and Response to Reviewers 30.01.docx [file pone.0343271.s007.docx]

Dear Academic Editor and Reviewers,

We sincerely thank the Academic Editor and the reviewers (1&2) for their careful assessment of our manuscript and for the constructive and insightful comments provided with minute details. We greatly appreciate the time and effort invested in reviewing our work, which has substantially strengthened the quality, clarity, and rigor of the manuscript.

The manuscript has been revised as follows:

- **Methodological Clarification:** Acknowledged the functional overlap between the cross-sectional audit and a retrospective cohort approach to clarify the epidemiological framework.
- **Generalizability:** Reinforced that findings specifically apply to high-mortality or high-risk programmatic settings (districts with >5% TB mortality).
- **Confounder Acknowledgment:** Justified the omission of BMI and clinical severity due to inconsistent secondary data and added cautionary notes to the Cox model interpretation.

We believe these refinements address the final minor corrections and thank you for your guidance.

We are grateful for the constructive guidance provided and hope that the revised version meets the expectations of the journal. We would be pleased to provide any further clarifications or revisions if required.

Thank you once again for your thoughtful review and consideration.

Sincerely,
Harsh Shah

(On behalf of all authors)

Dear Academic Editor,

We sincerely thank you for your detailed guidance and for the opportunity to revise our manuscript. We have carefully addressed each of the points raised and outline our responses below.

1. Compliance with PLOS ONE style and formatting requirements

We have revised the manuscript to fully comply with PLOS ONE style guidelines, including formatting of the main text, title page, author affiliations, tables, figures, and file naming conventions, in accordance with the official PLOS ONE templates provided. We have ensured consistency in font type, font size, line spacing, heading structure, reference style (Vancouver), and figure/table placement as per journal requirements.

2. Data sharing and Data Availability statement

We acknowledge the importance of data sharing and transparency. This study involved community-based verbal autopsies and includes sensitive human participant data such as detailed clinical timelines, health-seeking behaviours, and household-level socioeconomic information. Although all direct personal identifiers were removed prior to analysis, the combination of variables and small geographic units could permit indirect identification of participants.

Accordingly, ethical restrictions on public data sharing were imposed by the Institutional Ethics Committee of the Indian Institute of Public Health Gandhinagar, which approved the study. In line with PLOS ONE policy, we have updated the Data Availability statement to clarify that de-identified data can be made available upon reasonable request, subject to ethics approval and data protection requirements.

Requests for data access may be directed to corresponding author.

As such, option (b) does not apply, and the dataset has not been uploaded to a public repository due to these ethical constraints. The Data Availability statement in the submission system has been updated accordingly.

3. Supporting Information captions and citations

We have added captions for all Supporting Information files at the end of the manuscript, following PLOS ONE Supporting Information guidelines. All in-text citations referring to Supporting Information have been checked and updated to ensure accurate cross-referencing.

4. Reviewer-suggested citations

We carefully reviewed all publications suggested by the reviewers. Relevant references that strengthened the contextualization and interpretation of our findings have been incorporated into the revised manuscript. Citations that were not directly applicable to the study objectives or scope were not included, in line with editorial guidance.

We are grateful for these constructive suggestions, which have helped improve the clarity, rigor, and compliance of our manuscript. We hope that the revised version meets the journal’s requirements and are happy to provide any additional information if needed.

**Response to Reviewers**

Reviewers 1:

1. In study design, the justification for describing the study as a “cross-sectional audit with time-to-event analysis” is well explained. But for readers from epidemiology backgrounds, this study sounds as a retrospective cohort of deceased individuals. Consider briefly acknowledging this overlap explicitly in the Methods or Limitations to avoid conceptual confusion.
   - Thank you. We appreciate this insightful observation. We have added explicit acknowledgments of this methodological overlap in both the Study Design and Study Strengths and Limitations sections to provide conceptual clarity. (Line no. 84-88 and 377)
2. District selection was purposive and based on higher TB mortality (>5%), which is appropriate for identifying system bottlenecks. Authors need to reinforce that findings are most applicable to high-mortality or high-risk programmatic settings.
   - In earlier versions of manuscript, it has been addressed. However, we have reinforced the manuscript to explicitly state that the results are representative of high-risk or high-mortality programmatic environments, which may differ from lower-burden settings. (Line no. 372)
3. Important variables such as nutritional status (BMI), severity at diagnosis, and treatment of comorbidities could not be included. Justification for these omissions is needed and briefly reiterate this point when interpreting Cox model findings to prevent overinterpretation.
   - We have updated the Methods and results section to justify these omissions and the Discussion - limitation to caution against overinterpretation of the hazard ratios. (Line no. 172, 313 and 399-400)
4. Figure 1 is informative but dense. Please ensure that axis labels and legends are clearly readable in the final typeset version.
   - Noted. We will make sure during the type setting process to ensure to have axis labelled and good resolution image.
