# Supplementary material for: Factors accelerating time to death among persons with Tuberculosis in Western India: Evidence from a community-based retrospective death audit
Source: PLoS One. 2026 Feb 27;21(2):e0343271. doi: 10.1371/journal.pone.0343271 (PMC12948073; doi:10.1371/journal.pone.0343271)
Supplement: Attachment — Submitted filename: Reviewer Comments_reply.docx [file pone.0343271.s006.docx]

1.In study design, the justification for describing the study as a “cross-sectional audit

with time-to-event analysis” is well explained. But for readers from epidemiology

backgrounds, this study sounds as a retrospective cohort of deceased individuals.

Consider briefly acknowledging this overlap explicitly in the Methods or Limitations to

avoid conceptual confusion.

•Thank you. We appreciate this insightful observation. We have added explicit

acknowledgments of this methodological overlap in both the Study Design and Study

Strengths and Limitations sections to provide conceptual clarity. (Line no. 84-88 and

377).

**Reviewer:** Suggestions addressed very well.

2.District selection was purposive and based on higher TB mortality (>5%), which is

appropriate for identifying system bottlenecks. Authors need to reinforce that findings

are most applicable to high-mortality or high-risk programmatic settings.

•In earlier versions of manuscript, it has been addressed. However, we have reinforced

the manuscript to explicitly state that the results are representative of high-risk or highmortality programmatic environments, which may differ from lower-burden settings.

(Line no. 372)

**Reviewer:** Suggestions addressed very well.

3.Important variables such as nutritional status (BMI), severity at diagnosis, and

treatment of comorbidities could not be included. Justification for these omissions is

needed and briefly reiterate this point when interpreting Cox model findings to prevent

overinterpretation.

•We have updated the Methods and results section to justify these omissions and the

Discussion - limitation to caution against overinterpretation of the hazard ratios. (Line

no. 172, 313 and 399-400)

**Reviewer:** Suggestions addressed very well.

4.Figure 1 is informative but dense. Please ensure that axis labels and legends are

clearly readable in the final typeset version.

•Noted. We will make sure during the type setting process to ensure to have axis

labelled and good resolution image.

**Reviewer:** Suggestions addressed very well.
